# Pulmonary Embolism in Children

**DOI:** 10.3389/fped.2017.00170

**Published:** 2017-08-10

**Authors:** Ahmar Urooj Zaidi, Kelley K. Hutchins, Madhvi Rajpurkar

**Affiliations:** ^1^Division of Hematology Oncology, Carman and Ann Adams Department of Pediatrics, Wayne State University School of Medicine, Children’s Hospital of Michigan, Detroit, MI, United States

**Keywords:** pulmonary embolism, pulmonary artery thrombosis, children, deep venous thrombosis, pediatrics

## Abstract

Pulmonary embolism (PE) in the pediatric population is relatively rare when compared to adults; however, the incidence is increasing and accurate and timely diagnosis is critical. A high clinical index of suspicion is warranted as PE often goes unrecognized among children leading to misdiagnosis and potentially increased morbidity and mortality. Evidence-based guidelines for the diagnosis, management, and follow-up of children with PE are lacking and current practices are extrapolated from adult data. Treatment options include thrombolysis and anticoagulation with heparins and oral vitamin K antagonists, with newer direct oral anticoagulants currently in clinical trials. Long-term sequelae of PE, although studied in adults, are vastly unknown among children and adolescents. Additional research is needed in order to provide pediatric focused care for patients with acute PE.

## Introduction

Although first described almost two centuries ago by von Löschner (1), our knowledge of pediatric pulmonary embolism (PE) remains fragmented. These gaps in our knowledge are intensified by the infrequency of diagnosis of pediatric PE, thus limiting a standardized approach to investigative and management strategy. Hence, it is possible that the morbidity and mortality of undiagnosed PE in children may be underestimated. Historically, PE in children was thought to occur in the setting of infection, but it is becoming clear that PE is increasingly related to cancer, congenital heart disease, acquired and inherited thrombophilias, and central line placements (2). Early registries from Canada and the Netherlands providing national data indicate PE as a rare event among pediatric populations (3, 4). The incidence of venous pulmonary thromboembolism has been steadily increasing in children, as a consequence of longer survival of critically ill children, with conditions that predispose to thromboembolic disease, as well as the increased use of central venous catheters (5, 6).

Studies examining the incidence of PE in children report an incidence of 8.6–57 in 100,000 in hospitalized children, and 0.14–0.9 in 100,000 when studying the general population of non-hospitalized children (3, 7–10). The wide range of incidence in hospitalized children may be a manifestation of the often clinically silent nature of PE, misdiagnosis, more comprehensive reporting or a function of the biased population of a tertiary care center (7). The National Hospital Discharge Survey data from 1979 to 2001 yielded a population-based incidence of 0.49/10,000 patients/year (9). There appears to be a predilection of pediatric PE in infants and toddlers, with a second peak seen in teenagers (9). Black children are estimated to have an incidence 2.38 times higher than white children (9). However, it is likely that these numbers are underestimated due to the often silent nature of PE in children. This is corroborated by autopsy data, which shows discordant rates of PE based on clinical suspicion. In one study, the diagnosis was only considered in 15% of patients with PE (11). It is apparent that a high index of suspicion is mandated for timely and effective care for children with PE (12).

In this review, we aim to highlight the presentation, diagnostic work-up, treatment, risk factors, and follow-up of pediatric PE. We will also discuss emerging novel therapies and future directions of research in this field.

## Presentation

For almost one century, physicians have noted that PE may occur without the classic symptomatology among children (13). Unlike in adults, pediatric PE often appears clinically silent (2). On retrospective review of children with an eventual diagnosis of PE, however, symptoms or signs were often present but may have been missed resulting in misdiagnosis, such as pneumonia, exacerbation of heart failure, or malignancy (7, 10). The time to diagnosis of PE as compared to adults is often longer with mean time to diagnosis being as high as 7 days as reported in some studies (12). Therefore, keeping a high index of suspicion for PE in children is critical. The majority of cases in which an autopsy revealed PE did not have an ante-mortem diagnosis of PE (11). Classic symptoms when present include increased shortness of breath, pleuritic chest pain, hemoptysis, cough, and even syncope. Patients may present with tachycardia, tachypnea, and edema due to deep vein thrombosis (DVT), as well as signs of right heart failure (2, 14). In addition, patient symptoms may be thought to be related to other underlying medical conditions such as congenital heart disease or infection that often coincide and predispose the patient to PE, thus masking the diagnosis (2). This may often lead to delay in or misdiagnosis and potential additional serious consequences (7, 12, 14, 15). An underlying diagnosis of PE should be considered when patients are not improving on therapy especially in the setting of conditions known to predispose to PE (Table 1) (16). In adults, specific validated diagnostic prediction tools, such as the Wells’ criteria (17), the Geneva score (18), and the pulmonary embolism rule-out criteria (PERC) (19), exist for diagnosis of PE (20, 21). These models combine patient clinical signs and additional risk factors to assess pretest probability for the diagnosis of PE in adults (10). In children such models have not been validated. One study conducted by Biss et al. evaluated the modified Wells simplified probability score in 50 children with PE and 25 PE negative control patients, as well as D-dimer values in 27 PE positive and 12 PE negative patients and found that D-dimer had a low diagnostic utility for PE in children (22). Recently, a single-center retrospective study was conducted by Lee et al. in children undergoing either D-dimer testing or radiologic evaluation (computed tomography or ventilation-perfusion scan) in the emergency department setting. The investigators evaluated the test characteristics of the Wells criteria and PERC low-risk rule. Among the 561 patients, 36 (6.4%) were eventually diagnosed with PE. The Wells criteria demonstrated a sensitivity and specificity of 86 and 60%, respectively. The sensitivity and specificity of the PERC were 100 and 24%, respectively. A clinical decision rule, including the presence of oral contraceptive use, tachycardia, and oxygen saturation <95%, demonstrated a sensitivity and specificity of 90 and 56%, respectively, a positive and negative likelihood ratio of 2.0 and 0.2, and a positive and negative predictive value of 0.12 and 0.99, respectively (23).

**Table 1:** Risk factors to be considered in etiopathogenesis of pulmonary embolism (Virchow’s triad).

Damage to the endothelium Central venous catheters Inflammation (lupus, inflammatory bowel disease, etc.) Systemic infection Antiphospholipid antibodies Change in laminar flow Congenital or acquired heart disease Local anatomical causes (e.g., congenital anomalies of pulmonary arteries or after corrective heart surgery, e.g., Fontan surgery) Total parenteral nutrition Thrombophilia Acquired Nephrotic syndrome Cancer Medications e.g., l-asparaginase therapy Pregnancy or hormonal supplementation Antiphospholipid antibodies Inherited Deficiency of anticoagulants, e.g., protein S, C, and antithrombin III Factor V Leiden, prothrombin gene variant, etc. Elevated homocysteine

## Work-Up

Diagnostic tests for evaluation of PE can be divided into those needed for definitive diagnosis of PE (Table 2), tests that may aid in diagnosing the severity of PE (i.e., risk prediction and, thus, may help in decision making of management of PE) and miscellaneous tests that should be performed prior to anticoagulant therapy of PE.

**Table 2:** Advantages and disadvantages of diagnostic modalities and therapies.

Advantages and disadvantages of diagnostic tools and therapies in the management of pulmonary embolism in children		
**Diagnostic Tool**	**Advantages**	**Disadvantages**

Ventilation/perfusion scan	Safe and easy to perform	Low sensitivity False-positives from other diagnosis Difficult in younger patients Technically demanding

CT pulmonary angiography	Non-invasive Short study time Widely available Identifies alternate thoracic etiologies	May miss small peripheral emboli Radiation exposure, particularly in young females Contraindicated in renal insufficiency

Pulmonary angiography	Gold standard Generally diagnostic	Invasive Radiation exposure May not be easily available

Magnetic resonance imaging/magnetic resonance pulmonary angiography	No need for radiation or contrast Can assess cardiovascular anatomy	May miss small peripheral emboli Long duration of examination May not be easily available

**Therapy**	**Advantages**	**Disadvantages**

Unfractionated heparin (UFH)	Short half-life Reversal agent available	Continuous intravenous infusion Unable to administer outside of medical setting Possible development of heparin-induced thrombocytopenia (HIT) Frequent monitoring needed Risk of bleeding

Low molecular weight heparin	Easy to administer Reversal agent available	Effectiveness uncertain in obese patients Possible pain with administration Difficult to achieve therapeutic levels in infants Possible development of HIT (less than UFH) Risk of bleeding

Warfarin	Oral Able to monitor therapeutic level Reversible	Frequent monitoring Difficult to maintain in therapeutic window in children Multiple drug/food interactions Risk of bleeding

Direct oral anticoagulant	Oral No frequent blood draws	No way to monitor Few reversal agents Not approved for patients <18 years Risk of bleeding

### Tests for Diagnosis of PE

#### Ventilation/Perfusion (V/Q) Scan (Radionuclide Scintigraphy)

Ventilation/perfusion scans have historically been used to test for diagnosis of PE in children. While safe and easy to perform, they are not guaranteed to provide a definitive diagnosis. V/Q mismatch can be seen in pneumonia, sickle-cell disease, arterial stenosis, and air, fat, and foreign body embolism (24).

In general, this testing is done using the radiotracer 99m-labeled macroaggregated albumin. Areas clear of radiotracer activity represent reduced blood flow. To reduce the rate of false-positives, patients are supine during injection. Imaging, preferably in eight views (bilateral anterior and posterior oblique, anterior, posterior, and bilateral lateral), is then completed with the patient upright. At the very least, in critically ill patients, one anterior and bilateral oblique views must be used. Due to the need for active aerosol inhalation, these tests are technically difficult for younger patients (25).

Victoria et al. describe six patients out of thirteen with PE who underwent V/Q scans to determine its presence and describe four patients to have a positive result. The remaining two patients had low probability results (26). The Canadian registry shows that PE was diagnosed in 22 of 31 children who had a high probability V/Q scan (3). This test appears to be less favored when compared to CT pulmonary angiography (CTPA). The major limitation remains the fact that most patients have low or intermediate probability risk scans that are non-diagnostic (6).

#### CT Pulmonary Angiography

Due to its practicality, CTPA has rapidly overtaken V/Q scans as a primary imaging technique for diagnosis of PE. The speed, reliability, and ability to specifically detect other pathologies make this test ideal. In this modality, the criteria for acute PE include the presence of a sharply marginated complete or partial pulmonary arterial filling defect present on at least two consecutive images (25). Adult data show CTPA sensitivity of 83% (90% when done in combination with CT venography) and specificity of 95% (27). Similar data for/regarding children do not exist. Kritsaneepaiboon et al. (28) described a 9.3% false positive rate and a 2.4% false negative rate in pooled pediatric data from eight studies. The most critical disadvantages to this technique remain the exposure to ionizing radiation and insensitivity to small, sub-segmental emboli. New CT techniques are on the horizon to help mitigate some of these issues and increase the accuracy with reduced radiation. These include both imaging techniques, such as dual-energy CTA, and reconstruction algorithms, such as model-based iterative reconstruction and adaptive statistical iterative reconstruction. These reconstruction algorithms alleviate apprehension to radiation and are becoming more widely available. These reconstruction techniques provide the same anatomic detail as conventional scans (25).

#### Pulmonary Angiography

The traditional gold standard for diagnosis of PE, pulmonary angiography is invasive and expensive that limits its use in the pediatric population. This procedure includes weight-based injection of low-osmolar non-ionic contrast material through a pigtail catheter placed within the left or right pulmonary artery. The diagnosis is made when an intraluminal filling defect is recognized (25).

#### Magnetic Resonance Imaging/Magnetic Resonance Pulmonary Angiography (MRI/MRPA)

The elimination of ionizing radiation and use of safer contrast agents, make MRI/MRPA an attractive option for clinicians. Preliminary adult data show that MRI/MRPA may be a promising technique for those patients in whom CT is contraindicated (29). This topic is not extensively studied in children, so its effectiveness and reliability are uncertain in this patient population.

### Tests That May Aid in Risk Assignment

The clinical severity of PE varies widely. Children with PE may be asymptomatic (i.e., PE may be detected incidentally during other investigative work-up) or may present with complete cardiovascular collapse. While in some children, treatment with standard anticoagulation may be the appropriate treatment, in others, additional interventions such as thrombolysis or surgical thrombo-embolectomy may be warranted. Thus, after diagnosis of PE, an attempt should be made to categorize the patients into specific risk categories that can predict adverse outcomes in children with PE. In adults, specific models exist for risk prediction of PE and patients are often categorized into high risk (presentation with cardiovascular collapse), intermediate risk (patients who are normotensive but show evidence of right heart strain either on electrocardiogram [EKG], echocardiography, or by biomarkers) and low risk (symptomatic but absence of preceding features) (30, 31). Unfortunately, such risk categorization is not common in children. We suggest that the following investigations could be performed in children to aid in assessing the risk of PE. It should be noted none of these tests have been studied extensively in pediatrics for risk assignment.

#### Electrocardiogram

Electrocardiogram is fundamentally based on changes from cor pulmonale and subsequent right heart strain (2). This may show right axis deviation, right bundle branch block, sinus tachycardia, ST segment, and T wave abnormalities in adult patients (the classic S1Q3T3 pattern), but is not reliable or validated in pediatrics (32).

#### Echocardiogram

A 2D echocardiogram is an imaging modality that can examine both direct and indirect results of a PE. In adults, it allows for the ability to reasonably predict which patients are at risk for severe outcomes. These signs may include right ventricle dilatation, hypokinesis and abnormal motion of the interventricular septum, tricuspid regurgitation, and lack of collapse of inferior vena cava during inspiration. RV free-wall hypokinesis that does not affect the apical segment is highly specific, but not very sensitive (2, 33).

#### Biomarkers

Several biomarkers, such as cardiac troponin, brain-type natriuretic peptide, and heart type fatty acid-binding protein, have been shown in adults to increase the risk of adverse outcomes in PE and may be performed in children (18, 34, 35). The ranges of such biomarkers have not been established in children, and as such their clinical utility is uncertain.

### Other Ancillary Tests

Other ancillary studies that are usually performed include complete blood count with differential, prothrombin time, activated partial thromboplastin time, and fibrinogen level; renal and liver function tests should be performed prior to initiating anticoagulant therapy to assess any risk of bleeding. In addition, if pharmacologic therapy is being considered, a plasminogen level may be measured as neonates and children may often be deficient and supplementation with plasma may be needed to obtain the necessary therapeutic effect. Furthermore, clinicians should examine extremities and all four limbs should have ultrasonography to evaluate for any associated DVTs (36). Chest radiography, while not helpful in the diagnosis of PE, is very helpful in the exclusion of other lung pathologies.

#### Thrombophilia Testing

The Subcommittee of Perinatal and Pediatric Thrombosis of the Scientific and standardization Committee of the International Society on thrombosis and Hemostasis recommend that all children with thrombosis be tested for thrombophilia (as described in Table 1). However, the role of thrombophilia in categorization of risk, management, and outcomes has not been elucidated yet for pediatric PE (37).

## Treatment

Prompt recognition and diagnosis is of utmost importance to not only prevent progression and adverse sequelae of underlying PE but also to avoid unnecessary invasive treatment (38). Recommendations for management of the pediatric patients with PE have been extrapolated from adult data (2, 7, 39). Given the differences in pharmacokinetics and etiologies among children and adolescents compared to adults, however, management decisions should be specifically geared toward this particular population rather than simply based off of adult data (39).

**Figure 1:**
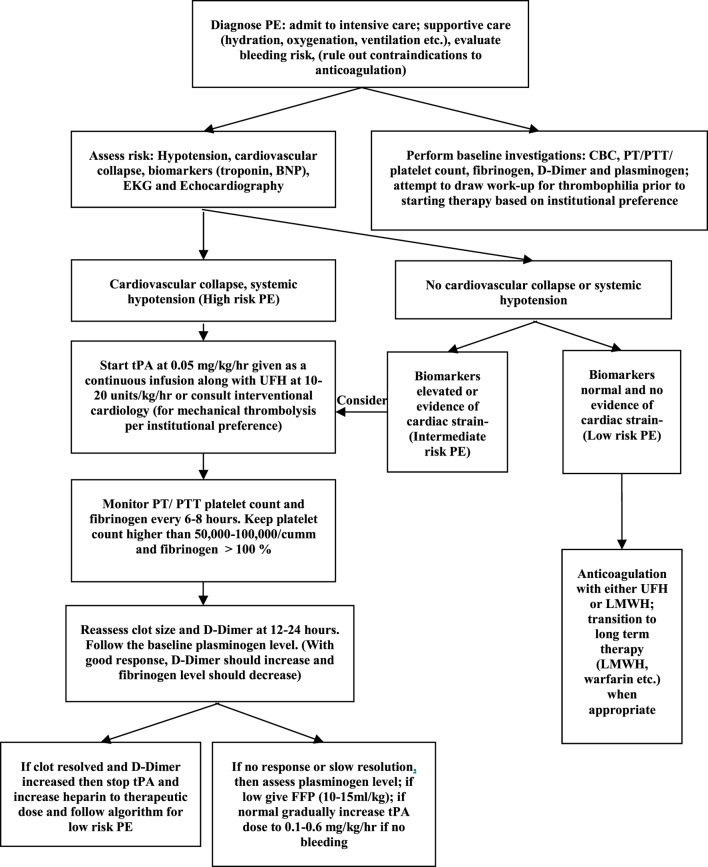
Treatment algorithm for pulmonary embolism.

There are no specific treatment algorithms for management of PE in children. Each institution should consider a treatment approach that works best for the individual setting. Some centers have developed pulmonary embolism response teams with involvement from multidisciplinary teams, such as hematology, emergency department, intensive care, and interventional cardiologists (31). Patients who present with signs and symptoms of high risk PE may benefit from either pharmacologic (Table 2) or mechanical thrombolysis. The goal of thrombolysis is to aim at a faster clot resolution, thus reducing right ventricular strain. Recombinant tissue plasminogen activator (rtPA; alteplase), streptokinase, or urokinase have been used for pharmacologic thrombolysis. Over the last several years, rtPA has been used more frequently due to its low immunogenicity, improved availability, *in vitro* clot lysis activity, and fibrin specificity (40). Thrombolysis may be delivered *via* a peripheral vein (systemic thrombolysis) or *via* catheter-directed thrombolysis where-in a catheter is placed in close proximity to the clot. Although there are no specific preferences, in general, catheter-directed therapy is preferred if there is a higher perceived risk of bleeding. Furthermore, although there are ACCP guidelines concerning dosing, the optimal dose for rtPA for the management of pediatric PE has not been established. Currently, there are two dosing regimens with each presenting potential advantage. One regimen uses rtPA at a higher dose of 0.1–0.6 mg/kg/h for 6 h. While this may offer the advantage of improved clot resolution, there may be higher risk of bleeding. On the other hand, a low-dose regimen consisting of 0.03–0.06 mg/kg/h with a maximum dose of 2 mg/h has been shown to be efficacious with less risk of bleeding (41). We present a treatment algorithm (Figure 1) that we follow at our center. In our dosing guideline, we start thrombolysis at a low dose and then escalate to a higher dose if no response is seen. Mechanical thrombolysis and surgical thromboendarterectomy may be utilized for patients with high risk of bleeding or with contraindications to anticoagulation, if pharmacologic treatment has failed or if the patient presents with hemodynamic collapse.

Unfractionated heparin (UFH: 75 U/kg over 10 min intravenous followed by 20 U/kg/h for patients >1 year, 28 U/kg/h for <1 year of age) and low molecular weight heparin (LMWH: 1 mg/kg twice daily subcutaneously for patients >2 months of age, 1.5 mg/kg twice daily subcutaneously for patients <2 months of age) remain the most commonly used initial therapies for children with PE. If the patient is not clinically stable or has a higher risk of bleeding, UFH may be used as this is easily reversible and has a shorter half-life. Long-term treatment consists of either LMWH or warfarin. The duration of therapy in PE has not been defined and treatment duration has varied from 3 months to 1 year. Newer direct oral anticoagulants such as rivaroxaban, which directly inhibits factor Xa, have been studied in randomized controlled trials among adults for use in PE (42), but their safety and efficacy have yet to be established among the pediatric population.

## Follow-Up

It is important to continue to follow pediatric patients in the acute setting of PE to determine the immediate outcome (resolution, progression, and recurrence) and to monitor for potential long-term complications, such as pulmonary hypertension and chronic PE (43). The sequelae of PE among children, however, are not well studied (7). Hancock et al. conducted a mixed retrospective-prospective cohort study of patients less than 21 years of age with PE (44). They studied 58 patients (47 of who were prospectively followed). Echocardiography was obtained in the acute period in 24 patients assessing the presence of right heart dysfunction. Four patients had one parameter suggestive of acute right heart dysfunction, five had two parameters, and one had all three. EKGs were obtained in 32 patients in the acute setting in which 13 had ST/T segment changes, four showed the classic S1Q3T3 pattern and two had voltage consistent with right ventricular hypertrophy. During the first 6-month follow-up period, 11 patients underwent echocardiography of whom two had tricuspid velocity >3 m/s, three had septal flattening, and four had right ventricular dilation. An additional 15 patients had echocardiography after 1 year of whom only 13% had septal flattening and right ventricular dilation. These results indicate that among the pediatric population with acute PE, acute cardiac dysfunction is relatively common but not in the chronic setting (44). Additional findings noted that five patients developed recurrent, symptomatic PE and there was a non-resolution of 18% among patients who underwent repeat imaging 6 months after the acute event.

## New/Emerging Concepts

For unstable patients, catheter-directed modalities are generating more interest, though pediatric data regarding the use and feasibility of these modalities are limited. Ultrasound-assisted thrombolysis (USAT) uses catheter-directed high frequency ultrasound to assist in penetration of a thrombolytic agent into the embolus. Two large adult studies have shown no difference in mortality or major bleeding between groups getting USAT with a fibrinolytic agent versus conventional anticoagulation (45, 46). Rheolytic embolectomy (the most common type being the AngioJet) injects pressurized saline into the embolus while aspirating macerated thrombus through the catheter port. Early adult data show promising results for patients with PE (47, 48). A major disadvantage of this procedure is that mandatory venotomy is required for insertion, which increases the bleeding risk. Rotational embolectomy uses a rotating device at the catheter tip that fragments the thrombus, in conjunction with continuous aspiration. In one adult study, 89% of patients with shock due to PE were stabilized (49). Suction embolectomy and thrombus fragmentation with rotation of a pigtail catheter or use of a balloon angioplasty catheter are also treatment modalities that are occasionally used in conjunction with USAT, rheolytic embolectomy, and rotational embolectomy. Perhaps the largest study in the pediatric population, studying 21 aspiration and rheolytic thrombectomies (5 of the pulmonary vasculature) at Texas Children’s Hospital, showed that such interventions can be performed safely even in critically ill children with life-threatening thrombosis (50).

## Future Research

Currently, there is an ongoing clinical trial to determine the optimal duration of treatment (6 weeks versus 3 months) for children with provoked DVT (51). This trial, however, does not allow enrollment of patients with PE. Prospective trials specifically addressing risk categorization and optimal treatment of pediatric PE are desperately needed.

## Conclusion

Pulmonary embolism is a rare, but potentially fatal, condition that often goes unrecognized among the pediatric population. It is critical to maintain a high index of suspicion of PE particularly among patients at greatest risk, including patients with a CVL, congenital heart disease or other conditions known to predispose to PE (obesity, hormonal supplementation, etc.). Diagnostic prediction models for the diagnosis of PE, such as the Wells criteria and Geneva score, have been validated among the adult population; however, there are no similar models for use among children and adolescents that are greatly needed. CT angiography is the primary modality utilized for diagnosis of PE in this age group. The mainstay of treatment remains UFH, LMWH, or warfarin for these patients, and trials are ongoing to determine the utility of newer oral anticoagulants as potential alternatives. It is critical that the field of pediatric hematology continues to focus research on patients with acute and chronic VTE not only to improve the knowledge and understanding of this disease process but to provide improved, evidenced-based care for these patients.

## Author Contributions

AZ and KH wrote the manuscript and contributed equally to the body of the text. MR revised the manuscript and generated key tables and figures. All persons who meet authorship criteria are listed as authors, and all authors certify that they have participated sufficiently in the work to take public responsibility for the content, including participation in the concept, design, analysis, writing, or revision of the manuscript.

## Conflict of Interest Statement

The authors declare that the research was conducted in the absence of any commercial or financial relationships that could be construed as a potential conflict of interest.
